# Public health round-up

**DOI:** 10.2471/BLT.20.011120

**Published:** 2020-11-01

**A stillbirth every 16 seconds**

A stillborn baby at the Princess Christian Maternity Hospital in Freetown, Sierra Leone, wrapped in cloth and awaiting burial by the family. Almost 2 million babies are stillborn every year according to the first ever joint stillbirth estimates released on 7 October. In 2019, an estimated 3 out of 4 stillbirths occurred in sub-Saharan Africa or Southern Asia.

**Figure:**
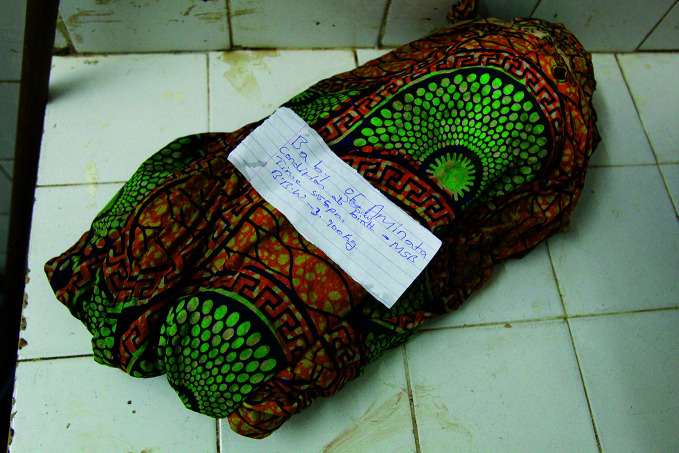
Asselin / UNICEF

## COVAX momentum

The COVAX Facility began signing formal agreements with novel coronavirus disease (COVID-19) vaccine developers and manufacturers to secure the doses needed to meet a one-billion-dose target that has been set for the end of 2021.

The COVAX Facility is the vaccine pillar of the Access to COVID-19 Tools (ACT) Accelerator, a global initiative that brings together governments, scientists, businesses, civil society, philanthropists and global health organizations (the Bill & Melinda Gates Foundation (BMGF), the Coalition for Epidemic Preparedness Innovations, the Foundation for Innovative New Diagnostics, Gavi, the Vaccine Alliance, the Global Fund, Unitaid, Wellcome, the World Health Organization (WHO), and the World Bank) to support the expedited development and equitable distribution of COVID-19 relevant diagnostics, vaccines and treatments.

As of 8 October, the COVAX Facility had brought together over 170 countries and economies, including 92 that are eligible for the COVAX Advance Market Commitment, a financing mechanism that will support accelerated access to safe and efficient vaccines as and when they are approved. China and the Republic of Korea are among the latest countries to join the initiative.

In order to meet the one-billion-dose target, the COVAX Facility has set an initial fundraising goal of US$ 2 billion by the end of 2020. As of 8 October, US$ 1.8 billion had been received in contributions and pledges from sovereign donors, the private sector, and philanthropic sources. According to Gavi, at least US$ 5 billion more is needed in 2021 to procure the doses required.

https://bit.ly/3lE6DX2

## COVID-19 diagnostic initiative

Partners in the ACT Accelerator agreed to make available 120 million quality, affordable COVID-19 antigen-based rapid diagnostic tests for low- and middle-income countries (LMICs).

A set of agreements were announced on 28 September, including volume guarantee agreements between the BMGF and test manufacturers Abbott and SD Biosensor which will price the tests at a maximum of US$ 5 per unit over a period of six months.

The Global Fund committed an initial US$ 50 million from its COVID-19 Response Mechanism to enable countries to purchase at least 10 million of the new tests. Arrangements are being made for the expedited market introduction of the tests in recipient countries with the support of the Africa Centres for Disease Control and Prevention, Unitaid and other partners.

WHO is providing policy guidance on the use of the tests and has granted Emergency Use Listing for two of them.

WHO guidance published on 11 September highlights the value of antigen-based rapid diagnostic tests in areas where community transmission is widespread and where nucleic acid amplification tests are either unavailable or where test results are significantly delayed.

https://bit.ly/2SNs1MX

## ACT Accelerator funding gap

The United Nations (UN) Secretary-General António Guterres reiterated his recent calls for increased investment in the ACT Accelerator. Convening a high-level event at the Seventy-fifth session of the UN General Assembly on 30 September, he said that it was in countries’ interest to work together to expand access to tests, treatments, and an eventual vaccine, and that the ACT Accelerator was the best mechanism for achieving those goals.

He stated that the US$ 3 billion so far committed (which includes the COVAX Facility funding mentioned above) has been critical for the start-up, but that the initiative requires an additional $ 35 billion, including an immediate infusion of $ 15 billion.

https://bit.ly/34SFsAX

## The pandemic’s multisectoral impact

The COVID-19 pandemic is presenting an unprecedented challenge to public health, food systems and the world of work. This is the core message of a joint statement issued by the International Labour Organization, the Food and Agriculture Organization, the International Fund for Agricultural Development and WHO on 13 October.

The statement outlines the economic and social disruption caused by the pandemic, which risks pushing tens of millions of people into extreme poverty and increasing the number of undernourished people (currently estimated at nearly 690 million) to 822 million by the end of the year.

The agencies stated that priority should be given to addressing underlying food security and malnutrition challenges, tackling rural poverty, extending social protection to all, facilitating safe migration pathways and promoting the formalization of work.

https://bit.ly/3dmWV8m

## Tuberculosis concerns

An estimated 10 million people fell ill with tuberculosis TB in 2019, the same number as reported in 2018. This is according to the *Global Tuberculosis Report 2020*, which was published on 14 October, and presents data on disease trends and the response to the epidemic in 198 countries and territories as well as an overview of pipelines for new TB diagnostics, drugs and vaccines. 

Key findings of the report include the fact that, despite modest progress in some areas – for example there has been an increase in access to treatment, with an estimated 7.1 million people accessing treatment in 2019, up from an estimated 7 million in 2018 – the overall picture is concerning, with the world as a whole, and many high burden countries, not on track to meet the 2020 milestones of the End TB Strategy. 

The report also presents data and projections regarding the impact of the COVID-19 pandemic on anti-tuberculosis campaigns, noting, for example, the sharp drop in case notifications reported in several high burden countries.

https://bit.ly/2H7lMkt

## Mental health services disrupted

The COVID-19 pandemic has disrupted or halted critical mental health services in 9 out of 10 countries worldwide.

This is according to a new WHO survey of 130 countries which was published on 5 October and provides the first global data on the impact of COVID-19 on access to mental health services.

Conducted from June to August 2020 across WHO’s six regions, the survey assessed COVID-19 related challenges impacting the provision of mental, neurological and substance use services, and what countries are doing to address them.

Among the report’s key findings was the fact that almost two thirds of the 130 countries reported disruptions to mental health services for vulnerable people, including disruptions to counselling and psychotherapy, critical harm reduction services, and opioid agonist maintenance treatment for opioid dependence.

The disruption occurs at a time of increased demand for mental health services, as the pandemic response measures exacerbate some mental health conditions.

https://bit.ly/2FoqhXw

## Tackling the infodemic

WHO, UN partners and the International Federation of Red Cross and Red Crescent issued a joint statement calling on countries to implement plans to tackle the problem of misinformation.

Issued on 23 September, the statement points out that while the media and social media are being used to keep people safe, informed, productive and connected, they are also enabling and amplifying an infodemic that is undermining global COVID-19 responses.

The statement calls on countries to promote the timely dissemination of accurate information based on scientific evidence to all communities, while respecting freedom of expression.

https://bit.ly/2SPFuEa

**Cover photo**

A team of health workers provide vaccination outreach services to remote villages in Fiji.

**Figure:**
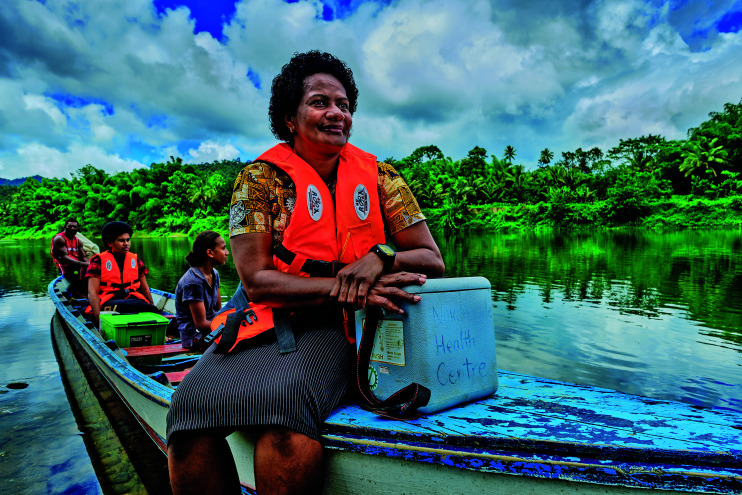
WHO / Yoshi Shimizu

## New stillbirth estimates

Almost 2 million babies are stillborn every year according to the first ever joint stillbirth estimates released by the United Nations Children’s Fund (UNICEF), WHO, the World Bank Group and the Population Division of the UN Department of Economic and Social Affairs.

Released on 7 October in a report titled, *A neglected tragedy: the global burden of stillbirths*, the estimates indicate that approximately 8 out of 10 stillbirths occur in low- and lower-middle-income countries (LMICs). In 2019, an estimated 3 out of 4 stillbirths occurred in sub-Saharan Africa or Southern Asia.

“Every 16 seconds, a mother somewhere will suffer the unspeakable tragedy of stillbirth,” said Henrietta Fore, UNICEF Executive Director, at the launch of the report, noting that most stillbirths could be prevented with proper monitoring and antenatal care and a skilled birth attendant.

The report warns that reductions in health services due to the ongoing pandemic could exacerbate the problem, projecting that a 50% reduction in such services could result in nearly 200 000 additional stillbirths over a 12-month period in 117 LMICs.

https://bit.ly/3lDYqlC

## Abuse allegations investigated

WHO Director-General Tedros Adhanom Ghebreyesus initiated an investigation of reported sexual exploitation and other abuse in the context of the ongoing Ebola virus disease response in the Democratic Republic of the Congo. A WHO statement released on 29 September condemned such abuses in the strongest terms and stated that anyone identified as being involved will be held to account and face serious consequences, including immediate dismissal.

https://bit.ly/2Io9YLn

**Looking ahead**

November 14 – World Diabetes Day. https://bit.ly/3dofitG

November 15 – World Day of Remembrance for Road Traffic Victims. https://bit.ly/34YQSDw

November 18–24 – World Antimicrobial Awareness Week. https://bit.ly/3iUSgM7

